# Characterization of Carbonyl Compounds in the Ambient Air of an Industrial City in Korea

**DOI:** 10.3390/s110100949

**Published:** 2011-01-17

**Authors:** Young-Kyo Seo, Sung-Ok Baek

**Affiliations:** Department of Environmental Engineering, Yeungnam University, Gyeongsan 712-749, Korea; E-Mail: youngkyo@ynu.ac.kr

**Keywords:** acetaldehyde, formaldehyde, VOCs, odorants, electronic industry

## Abstract

The purpose of this study was to characterize spatial and temporal variations of carbonyl compounds in Gumi city, where a number of large electronic-industrial complexes are located. Carbonyl samples were collected at five sites in the Gumi area: three industrial, one commercial, and one residential area. Sampling was carried out throughout a year from December 2003 to November 2004. At one industrial site, samples were taken every six days, while those of the other sites were for seven consecutive days in every season. Each sample was collected for 150 minutes and at intervals of three times a day (morning, afternoon, and evening). A total of 476 samples were analyzed to determine 15 carbonyl compounds by the USEPA TO-11A (DNPH-cartridge/HPLC) method. In general, acetaldehyde appeared to be the most abundant compound, followed by formaldehyde, and acetone+acrolein. Mean concentrations of acetaldehyde were two to three times higher in the industrial sites than in the other sites, with its maximum of 77.7 ppb. In contrast, ambient levels of formaldehyde did not show any significant difference between the industrial and non-industrial groups. Its concentrations peaked in summer probably due to the enhanced volatilization and photochemical reactivity. These results indicate significant emission sources of acetaldehyde in the Gumi industrial complexes. Mean concentrations of organic solvents (such as acetone+acrolein and methyl ethyl ketone) were also significantly high in industrial areas. In conclusion, major sources of carbonyl compounds, including acetaldehyde, are strongly associated with industrial activities in the Gumi city area.

## 1. Introduction

In a large urban area, volatile organic compounds (VOCs), including carbonyl compounds (CCs), can be emitted from a variety of emission sources such as motor vehicles, gas stations, laundries, cooking, and heating *etc.* [1–4]. The sources of VOCs in industrial areas are expected to be different from those in urban areas due to fugitive emissions of raw materials and organic solvents. Therefore, the occurrence patterns of VOCs in a particular industrial area are often found to be different from those of normal urban areas [5].

The Gumi National Industrial Complex (GNIC) was established as the nation’s largest inland high technology export base by the Korean government’s export-oriented policy in the early 1970s. The major industrial sectors of the city were developed focusing on electronic and semiconductor industries. In early days, textiles or household electronic appliances were dominant, which later gave way to semiconductor and digital industries (such as LCD/LED and cell phone manufacture) as leading sectors of the complexes [6].

The GNIC consisted of three sub complexes. The first complex, built in 1973 on an area of 10.2 km^2^, houses mainly textiles, electronics, and other miscellaneous industries. The second complex, with an area of 2.3 km^2^, was completed in 1983 to primarily accommodate semiconductor and electronics industries. The third complex, with an area of 5.1 km^2^, was completed in 1992 with high tech industries. The businesses housed in the whole industrial estate were 1,227 as of December 2009, including 392 electrical and electronic industries and 442 machinery industries. These two sectors thus account for the majority of the industries housed therein [7,8]. Unlike the steel or dyeing industry, the electronic industry is occasionally classified as an environmentally-friendly one, as it consumes very little fossil fuels or industrial water. However, a variety of organic solvents are used in large quantities in the LCD or semiconductor manufacturing process in particular, making the Gumi area likely to cause VOC contamination problems [5].

According to the data released by the Korea Ministry of Environment, a total of 51 cases of public complaints for foul smells were recorded in the city in 2006, 11 of which were associated with the industrial complexes [9]. Besides, the recent media coverage showed that the public complaints regarding foul smells have been filed consistently against the industrial complexes in particular since two to three years ago [10]. Nevertheless, the Gumi industrial complexes have not yet been under special surveillance (as sources of foul smells), which is currently set to cover 20 areas in nine cities in Korea. At present, a total of 22 foul-smelling odorants are listed in the Malodor Prevention Act in Korea [11]. Among them, six compounds are classified as CCs, including aldehydes and ketones, which occupy a comparatively large portion.

CCs are formed principally by photochemical reactions, while they are also emitted directly into the atmosphere from such sources as incomplete combustion of materials (of biological resources) or fossil fuels. They can also be released via fugitive emissions from industrial manufacturing processes, exhaust gases emitted from motor vehicles and so forth [12–16]. In particular, formaldehyde and acetaldehyde among many CCs are highly important, as they simultaneously belong to the 35 species of hazardous air pollutants in Korea [17]. Formaldehyde is well-known as a Group I carcinogenic substance listed by the IARC [18], whereas acetaldehyde is grabbing a great deal of public attention as a foul-smelling substance [11,14]. This study aimed to characterize the behavior of CCs in the atmosphere of Gumi city, to investigate the major controlling factors on the distribution of CCs in the city, and to provide scientific information needed in mapping out an appropriate air quality management measure for the GNIC, where large-scale electronic industries have been established.

## 2. Materials and Methods

### 2.1. Sampling Sites and Periods

**Figure 1.**
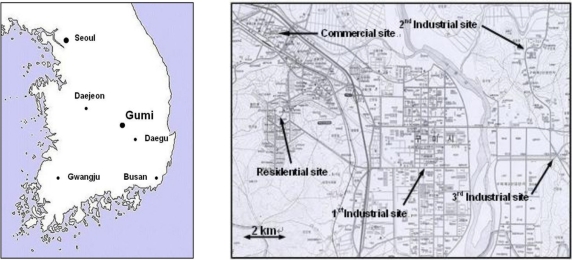
Locations of Gumi city and five sampling sites in the city.

The locations of Gumi city in Korea and sampling sites selected in the city are shown in Figure 1. In this study, a total of five sampling sites were selected, three industrial areas, one residential, and one commercial area. The first site was located in the first industrial complex at about 10 m from the ground, on the top of a three-story building. The second site was located in the second industrial complex, while the third site was in a complex (industrial, commercial and residential) zone. The fourth and the fifth site were located in a traditional commercial area and a newly developed residential area, respectively. All the sampling equipments were placed on the top of three or four-story buildings (about 10 to 14 m from the ground).

Sampling started from December 2003 and finished in November 2004. The first industrial site was selected as an intensive sampling site, as it was expected to be affected by strong sources. Sampling at this site was carried out every six days in light of its environmental significance, whereas at the other four sites measurements were conducted during seven consecutive days in each of four seasons. In each sampling day, three samples were collected (*i.e.*, morning, afternoon, and evening). Consequently, a total of 476 samples were collected in this study. The environmental conditions during the sampling are summarized in Table 1.

### 2.2. Sampling and Analytical Methods

In this study, a total of 15 CCs were determined. For their qualitative and quantitative analyses, a carbonyl-DNPH mixture (Supelco Inc, USA) was calibrated by preparing working standards through a five-step dilution process, *i.e.*, 0.25, 0.5, 1.0, 3.0 and 15.0 μg/mL (in case of formaldehyde). Methyl ethyl ketone, which was not included in the commercial standard mixture, was calibrated separately from the mixture, using an individual standard after dilution. Sampling of CCs was conducted using DNPH cartridge/cartridges (Supelco Inc., USA). To remove any possible interference from ozone, a polypropylene tube filled with KI (about 1.4 g) was connected to the front of the DNPH cartridge. Typically, 150 L of air was sampled at 1 L/min for 150 minutes using a pump, and the samples were kept under refrigeration before solvent extraction. DNPH derivatives were extracted with 3 mL of acetonitrile, and the extracted solution was transferred to a brown vial, tightly sealed with a Teflon tape, and stored under refrigeration. All the glassware was pre-washed with acetonitrile and dried at 60 °C (or over) before use. The glassware and extracted solutions were kept in such way that exposure to the air should be minimized. The extracted DNPH derivatives were analyzed by using HPLC with UV detection at 360 nm [19]. For a stationary phase, a C_18_ column was used with a mobil phase consisting of acetonitrile and water/acetonitrile/tetrahydrofuran. The operating conditions of HPLC used in this study are presented in Table 2. Because the peaks of acetone and acrolein were not separated by the HPLC, they were regarded as a group and expressed as such.

### 2.3. Quality Control and Quality Assurance

Repeatability of the analytical method was evaluated by means of a relative standard deviation (RSD) of the peak areas for a standard mixture (0.25 μg/mL level). As a result, a within-a-day analytical repeatability appeared to be less than 3.5%, while between-days repeatability less than 8.0%. The method detection limit (MDL) for each of target analytes was evaluated according to the U.S. EPA guideline [20] by analyzing a low level standard mixture 10 times repetitively. As a result, the MDL values for target analytes were estimated to be 1.1∼2.3 ng/mL, which are equivalent to 0.01∼0.02 ppb as concentrations in the air based on typical sampling volume of 150 L.

## 3. Results and Discussion

### 3.1. Occurrence and Distributions of Carbonyl Compounds

In this study, a total of 476 samples were collected at five sites, representing industrial, commercial, and residential areas. Detection frequencies for various CCs and their concentrations are summarized in Table 3. In general, formaldehyde and acetaldehyde are known to be the most abundant CCs in the urban atmosphere [14,16]. In this study, the detection frequency of these compounds appeared to be 100%, as they were measured in all the samples. Acetone used as a solvent (including a trace of acrolein) and methyl ethyl ketone were also measured at 99% or over, demonstrating that such substances are widespread in the industrial area. Detection frequencies of other substances such as propionaldehyde, crotonaldehyde, *i*-valeraldehyde, butyraldehyde were 76, 66, 59, and 49%, respectively. However, *n*-valeraldehyde, tolualdehyde, hexaldehyde, and 2,5-dimethylbenzaldehyde were below detection limits in all samples.

The mean concentrations (in ppb) of CCs for the total data are arranged in the descending order of acetaldehyde (4.44), formaldehyde (3.55), acetone+acrolein (3.31), methyl ethyl ketone (2.74), and propionaldehyde (0.97). On the contrary, the mean values of crotonaldehyde, butyraldehyde and *i*-valeraldehyde were 0.31 ppb or less, demonstrating that they exist at very low concentrations in the ambient atmosphere. Among those CCs, acetaldehyde and acetone are largely emitted from industrial manufacturing processes, while formaldehyde is commonly derived from both indoors and outdoors. Methyl-ethyl-ketone, which is sometimes used in the industrial setting as well, is known to be emitted from motor vehicles, landfills, and so forth as a combustion product [16]. Accordingly, such CCs are apparent substances closely related with anthropogenic/human activities.

### 3.2. Spatial Variations of Carbonyls Concentrations

**Figure 2.**
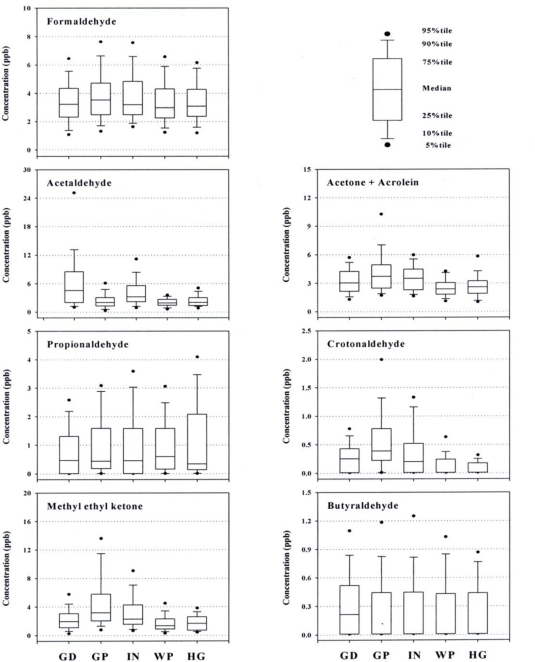
Comparison of concentration distributions for each sampling site; GD: the 1st industrial site; GP: the 2nd industrial site; IN: the 3rd industrial site; WP: commercial site; HG: residential site.

Spatial variations of selected CCs are presented in Table 4 (and Figure 2). CCs of environmental concern with the highest level varied from site to site, indicating the effects of local emission sources. In the 1st and the 3rd industrial site, acetaldehyde was predominant, while methyl ethyl ketone was on the top in the 2nd industrial site. However, in non-industrial (commercial and residential) areas, formaldehyde was the highest as expected. Although an average concentration of formaldehyde was measured as the highest in the 2nd industrial site, there was no significant difference in its concentrations between the sites. It is most likely because formaldehyde, being emitted from a variety of sources (such as motor vehicles, indoor furniture, insulation materials, and combustion [21]), while heating and cooking is present all over the Gumi area, regardless of locations selected for the sampling.

As shown in Table 4, the average concentration of acetaldehyde, a specified hazardous air pollutant as well as a designated foul-smelling substance in Korea [17], marked 7.35 ppb in the 1st industrial site, a level 2 to 3 times higher than elsewhere, with the maximum concentration of 77.7 ppb ever detected or approximately 10 times as high as the average concentration. Article 8, Paragraph 1 of the Foul Smell Prevention Act in Korea has set the maximum allowable concentration of acetaldehyde at 100 ppb on the border of the emission source in an industrial area and 50 ppb elsewhere [11]. The sampling site was on the top of a building hundreds of meters away from emission sources. This makes it most likely that the concentration along the line bordering the source of emission should easily exceed the maximum allowable level, and that the public complaints associated with foul smells filed occasionally in this city are also related with acetaldehyde. CCs related with organic solvents (like acetone+acrolein and methyl ethyl ketone) are measured fairly high in and around all the industrial sites compared to those in commercial and residential sites. This is most likely because such compounds are affected directly or indirectly by industrial activities. In addition, relatively high levels of methyl ethyl ketone in the 2nd and 3rd industrial sites indicate that these sites, both located next to main streets, are supposedly affected by combined impacts from industrial activities and vehicle exhausts since this compound is also emitted from motor vehicles as a combustion product.

In order to investigate the impacts of industrial activities in relation to the observed concentrations of CCs, the data were divided into two groups, *i.e.*, industrial (three sites) and the non-industrial (two sites) groups. Results of the two data groups are shown in Table 5. Formaldehyde did not show any significant difference between the two groups, while acetaldehyde from industrial data group appeared to be approximately 2.5 times higher. In the meantime, methly ethly ketone was 1.8 times higher than in the non-industrial group, suggesting direct or indirect sources of such hazardous substances in the industrial areas. In fact, methyl ethyl ketone is reported to be used as a solvent in the chemical and/or non-metallic sector in the Gumi Industrial Estate, in line with the evidence of industrial impacts [22]. However, acetaldehyde is not commonly used as an organic solvent or as a raw material in the industry. Thus, it is estimated that acetaldehyde is largely emitted during manufacturing processes or conversions of primary or secondary alcohols used in the industrial sources [12,13].

### 3.3. Temporal Variations of Carbonyl Concentrations

**Figure 3.**
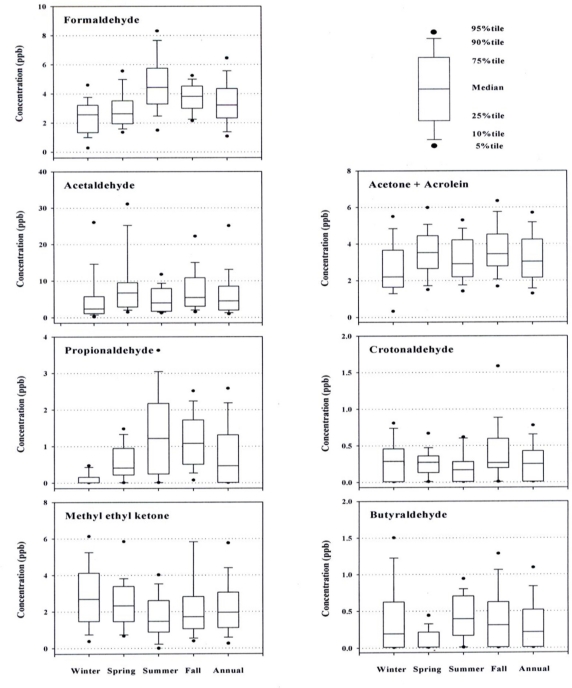
Seasonal concentrations of carbonyl compounds in the 1st industrial site.

Seasonal variations of carbonyl compounds do not appear to be greatly different from each other. Therefore, seasonal data for the 1st industrial site, which showed the highest levels in most cases, are illustrated in Figure 3, as a typical example. Formaldehyde, a substance detectable everywhere in the ambient air indoors or outdoors, turned out to be the highest in summer and the lowest in winter, showing a typical ‘high-in-summer and low-in-winter’ pattern reported elsewhere [14,16,23,24]. The increased concentration of formaldehyde in summer is attributable not only to the increased volatile emissions (due to higher temperature), but to the formation of secondary pollutants as a byproduct of photochemical reactions in summer [12,24,25]. Concentrations of the other carbonyl compounds such as acetaldehyde, methyl ethyl ketone, and acetone+acrolein did not noticeably vary across seasons, indicating these compounds are largely associated with local emission sources with a variety of independent and irregular industrial activities.

In this work, to investigate any variations in CC levels during a day, samples were taken three times daily, *i.e.*, in the morning (9 to 11 a.m.), afternoon (2 to 4 p.m.), and evening (7 to 9 p.m.). Concentrations of carbonyl compounds during a day varied between sites and compounds, making it difficult to make an arbitrary determination. However, it was noticed that the concentrations of most CCs, except formaldehyde, generally rose in the morning, fell in the afternoon and re-rose again in the evening. The rise and fall in the CC levels during a day is supposedly attributable not only to the atmospheric stability, but also to traffic volumes in the Gumi area. In general, the traffic volume increased in the morning and the evening. In contrast, the mixing height becomes relatively increased in the afternoon compared to the morning and the evening due to rise in temperature [16,23,25]. In this study, the highest concentration (77.7 ppb) for acetaldehyde was measured in an industrial site during the evening hour. However, the concentrations of formaldehyde rose during the afternoon time particularly in summer in most cases. This may well support the fact that there is a contributive portion of the secondary formation of formaldehyde through photochemical reactions in the air [12,24].

### 3.4. Comparisons of Gumi Data with Other Cities in Korea

Because the emission sources of CCs vary widely, the occurrences and concentrations of each compound in a city also vary according to the features characterizing the urban area. In this context, carbonyl concentration data measured in other cities or industrial complexes throughout the country were compared with those measured in this study, and the results are summarized in Table 6.

When the concentrations of formaldehyde in the industrial area are considered, those of the Gumi area are relatively low compared with other cities. However, the concentrations of acetaldehyde at the 1st industrial site stayed approximately three times as high as those in Siheung, Ansan, Yeosu, and Gwangyang cities, where a large scale of national industrial complexes have been established. Even at the 3rd industrial site in Gumi, the figure turned out about twice the level of those from the foregoing cities. As shown in Table 6, concentrations of formaldehyde are generally greater than those of acetaldehyde in many other urban and industrial areas in Korea. However, it is striking to find that the concentration of acetaldehyde in the 1st industrial complex in Gumi city is much higher than formaldehyde. Overall, concentrations of acetone+acrolein were high, compared with Siheung, Ansan, and Gwangyang. Our results also indicate that in the case of methyl ethyl ketone at the 2nd industrial site of Gumi was higher than those of Yeosu and Gwangyang [28], while lower than Ansan [27]. Propionaldehyde, another foul-smelling compound, also showed relatively higher levels in the Gumi area than other industrial and urban areas in Korea. On the contrary, CC levels in non-industrial areas of the Gumi city were not so different from those in other cities. Accordingly, since evidently most at issue is acetaldehyde at the 1st industrial complex of the Gumi city, an urgent and intensive attention to this matter is in order.

### 3.5. Correlations of Carbonyl Compounds and Air Quality/Meteorological Data

To investigate the factors affecting the CCs in the study area, correlation analysis was carried out using air quality and meteorological data. The air quality data of criteria pollutants such as SO_2_, PM_10_, O_3_, NO_2_, and CO were obtained from the nationwide ambient air monitoring network. There are three monitoring stations in the Gumi city, which cover the 1st industrial site, a residential, and a commercial site. For statistical analysis, all the air quality and meteorological data were re-calculated to have the same averaging time as carbonyl compounds. The results of correlation analysis (as Pearson coefficients) are shown in Table 7. In the case of the industrial site, correlation coefficient did not run up very high, while SO_2_ and NO_2_ exhibited significant correlations with some CCs. In addition, the correlation coefficients for acetone+acrolein and methyl ethyl ketone at the industrial site appeared to be 0.40, a moderate level. At the commercial site, the correlation coefficients of methyl ethyl ketone with PM_10_ and NO_2_ were 0.49 and 0.44, respectively, levels relatively high, due most likely to exhaust gases from motor vehicles.

Overall, however, the correlations of carbonyl compounds with combustion related criteria pollutants such as SO_2_, NO_2_, and CO did not show any distinctive relationships each other, implying that most of carbonyl compounds are largely associated with fugitive emissions instead of combustion related sources. The correlation coefficients with formaldehyde and ambient temperature were in the range of 0.30 to 0.54, a level relatively high, thus confirming that formaldehyde can be a secondary product through photochemical reactions as well as an ozone precursor. In this context, it might be expected that formaldehyde should be correlated positively with ozone, particularly during the summer season. According to Table 7, where all the four season data were included in the statistical analysis, the correlation coefficients between formaldehyde and ozone appeared to be very low. However, when only summer data were considered, the coefficients between the two variables appeared to be positively high (*i.e.*, 0.70, 0.55, and 0.34 for the residential, commercial, and industrial site, respectively).

Unlike other sites, levels of both acetaldehyde and methyl ethyl ketone at the industrial site turned out to be nearly not or negatively correlated with the temperature. It is thought that in view of the characteristics of the industrial area, these compounds are affected more effectively by the amount of raw materials used in the industrial processes rather than seasonal influence. As expected, wind speed and CCs turned out to be inversely correlated, due to the dilution effect, whereas relative humidity and other meteorological parameters did not show any clear patterns.

Theoretically, if a carbonyl compound has high correlations with other compounds, they might be considered to be affected by same sources. Correlation coefficients between different CCs were included in Table 8. One notable thing is that at the 2nd industrial site there was a high correlation between formaldehyde and acetaldehyde (0.85), and moderate correlations between these two compounds and propionaldehyde (0.53 and 0.47). It is also interesting to note that there is a large municipal landfill site near the 2nd industrial site. Therefore, odor related carbonyls at this site seem to be strongly affected by the presence of the landfill. Overall, correlations between acetaldehyde and other carbonyls were varied from site to site with no clear pattern, indicating that acetaldehyde in Gumi city are associated with various local industrial sources.

## 4. Conclusions

In this study, a total of 476 ambient air samples were collected in Gumi city in Korea, where the nation's largest inland high-tech industrial complexes have been established. In general, acetaldehyde appeared to be the most abundant CC, followed by formaldehyde, and acetone+acrolein. Mean concentrations of acetaldehyde appeared to be 2∼3 times higher in industrial sites than in other residential and commercial sites, with the highest concentration of 77.7 ppb. This finding thus suggests that it is most likely the primary cause of reported foul smells in the Gumi area. The ambient levels of acetone+acrolein and methyl ethyl ketone used as organic solvents were also higher in the industrial area than in the commercial and residential areas, indicating that they are affected directly or indirectly by the industrial establishments that use these solvents. In contrast, ambient levels of formaldehyde did not show any significant difference between the industrial and non-industrial data groups. Concentrations of formaldehyde in summer were higher than other seasons, due probably to the enhanced volatilization emissions and photochemical reactivity. However, other carbonyls including acetaldehyde did not show a specific seasonal pattern, presumably because they are locally affected by specific sources of emission within the industrial complexes. A comparison of CC concentration levels measured in the Gumi area with those of other major industrial areas in Korea revealed that the concentration of acetaldehyde in an industrial area of Gumi City was approximately three times higher than elsewhere. Therefore, our findings demonstrate that it is necessary to designate acetaldehyde at the 1st industrial complex, in particular, of Gumi City as one of the top priority pollutants earmarked for intensive supervision and surveillance. Because acetaldehyde is presumed to be emitted as a by-product through industrial manufacturing processes rather than used as a solvent, it is often overlooked or omitted in the emission inventory. Thus, direct on-site investigations and actual measurements for this compound are necessary along with appropriate process control with a management protocol. As acetaldehyde has been listed as one of hazardous air pollutants, and also specified as an odor-emitting compound by the Korean Ministry of Environment, it should be controlled more carefully by the existing regulation.

## Figures and Tables

**Table 1.** Sampling periods and meteorological conditions for each site.

**Sampling Site**	**Season**	**Sampling Period**	**Meteorological Condition**
1st industrial site	yearly	2003.12.1∼2004.11.30 (every 6 day)	15.8 ± 9.9 °C, clear and rainy
Other sites	seasonally	Winter: 2004.1.13∼1.19	1.6 ± 2.5 °C, clear and snowy
		Spring: 2004.4.9∼4.15	20.2 ± 3.2 °C, clear
		Summer: 2004.8.13∼8.19	26.2 ± 3.9 °C, occasionally rainy
		Fall: 2004.10.15∼10.21	17.1 ± 3.8 °C, clear and cloudy

**Table 2.** Operating conditions of HPLC for carbonyl compounds analysis.

**Operating Parameter**	**Specifications and Conditions**
HPLC System	Shimadzu SCL-6B with Shimzdzu SPD-6AV UV/VIS detector at 360 nm
Analytical column	Shim-Pack CLC-ODS (M) 4.6 mm × 150 mm with a C_18_ guard column
Mobile phase	A: acetonitrile 100 (V), B: water/acetonitrile/tetrahydrofuran 50/45/5 (v/v)
Gradient elution	100% B for 5 min, and then 60:40% A:B for 15 min, finally 100% A for 5 min
Flow rate and injection volume	1.0 mL/min and 20 μL injection

**Table 3.** Statistical summaries of carbonyl compounds along with detection frequencies (n = 476).

**Compound**	**Detection Frequency**	**Mean (ppb)**	**S.D. (ppb)**	**Median (ppb)**	**Min. (ppb)**	**Max. (ppb)**	**μg/m^3^ per ppb ^(2)^**
Formaldehyde	100%	3.55	1.78	3.22	0.14	12.45	1.34
Acetaldehyde	100%	4.44	6.73	2.61	0.20	77.71	1.97
Acetone/Acrolein	100%	3.31	1.84	2.96	< LDL. ^(1)^	15.61	2.59/2.50
Propionaldehyde	76%	0.97	1.15	0.46	< LDL.	7.25	2.59
Crotonaldehyde	66%	0.31	0.43	0.25	< LDL.	4.12	3.13
Methyl ethyl ketone	99%	2.74	2.78	2.04	< LDL.	28.13	3.22
Butyraldehyde	49%	0.28	0.38	< LDL.	< LDL.	2.52	3.22
*i*-Valeraldehyde	59%	0.20	0.25	0.10	< LDL.	1.57	3.85

(1) less than a lower detection limit (LDL);

(2) based on 0 °C, 1 atm.

**Table 4.** Concentrations (ppb) of carbonyl compounds at each sampling site in Gumi city.

**Sites**	**Carbonyl Compound**	**Mean**	**S.D.**	**Median**	**Max.**	**Min.**
1st industrial site (n = 172)	Formaldehyde	3.40	1.66	3.22	9.10	0.14
	Acetaldehyde	7.35	10.19	4.55	77.71	0.22
	Acetone+Acrolein	3.28	1.56	3.04	11.06	< LDL.
	Propionaldehyde	0.80	0.90	0.47	4.11	< LDL.
	Crotonaldehyde	0.31	0.31	0.25	2.06	< LDL.
	Methyl ethyl ketone	2.40	1.97	1.96	12.98	< LDL.
	Butyraldehyde	0.33	0.41	0.21	2.52	< LDL.

2nd industrial site (n = 78)	Formaldehyde	3.87	1.90	3.53	10.31	0.46
	Acetaldehyde	2.52	1.90	2.02	9.72	0.20
	Acetone+Acrolein	4.27	2.70	3.72	15.61	1.29
	Propionaldehyde	1.01	1.18	0.44	6.19	< LDL.
	Crotonaldehyde	0.61	0.69	0.39	4.12	< LDL.
	Methyl ethyl ketone	4.81	4.74	3.18	28.13	0.24
	Butyraldehyde	0.26	0.38	< LDL.	1.45	< LDL.

3rd industrial site (n = 77)	Formaldehyde	3.86	2.01	3.19	12.45	1.23
	Acetaldehyde	4.22	3.22	3.24	15.69	0.21
	Acetone+Acrolein	3.59	1.49	3.52	9.07	1.12
	Propionaldehyde	1.04	1.36	0.46	7.25	< LDL.
	Crotonaldehyde	0.36	0.44	0.20	1.85	< LDL.
	Methyl ethyl ketone	3.32	2.64	2.28	13.28	< LDL.
	Butyraldehyde	0.25	0.40	< LDL.	1.59	< LDL.

Commercial site (n = 74)	Formaldehyde	3.36	1.62	2.97	7.85	1.08
	Acetaldehyde	2.05	0.95	1.94	5.45	0.33
	Acetone+Acrolein	2.68	1.63	2.39	13.38	0.76
	Propionaldehyde	1.03	1.12	0.60	5.61	< LDL.
	Crotonaldehyde	0.17	0.30	< LDL.	2.09	< LDL.
	Methyl ethyl ketone	1.71	1.25	1.35	6.75	0.21
	Butyraldehyde	0.26	0.36	< LDL.	1.44	< LDL.

Residential site (n = 75)	Formaldehyde	3.46	1.77	3.08	11.10	1.01
	Acetaldehyde	2.34	1.21	2.03	5.43	0.23
	Acetone+Acrolein	2.74	1.36	2.59	7.45	0.80
	Propionaldehyde	1.21	1.39	0.34	5.33	< LDL.
	Crotonaldehyde	0.10	0.12	< LDL.	0.65	< LDL.
	Methyl ethyl ketone	1.80	1.12	1.66	4.79	< LDL.
	Butyraldehyde	0.25	0.31	< LDL.	1.09	< LDL.

**Table 5.** Comparison of carbonyl concentrations (in ppb) in industrial and non-industrial areas.

**Compounds**	**Industrial area (Mean ± S.D., n = 327)**	**Non-industrial area (Mean ± S.D., n = 149)**	**Mean ratio (Industrial to non-industrial area)**
Formaldehyde	3.62 ± 1.82	3.41 ± 1.69	1.06
Acetaldehyde	5.46 ± 7.88	2.20 ± 1.10	2.48 *
Acetone+Acrolein	3.59 ± 1.92	2.71 ± 1.50	1.33 *
Propionaldehyde	0.91 ± 1.09	1.12 ± 1.26	0.81
Methyl ethyl ketone	3.19 ± 3.15	1.76 ± 1.19	1.82 *
Butyraldehyde	0.30 ± 0.40	0.26 ± 0.34	1.16

* Industrial and non-industrial groups are significantly different at a level of 0.05 by Mann-Whitney test.

**Table 6.** Ambient concentrations (ppb) of carbonyl compounds in urban and industrial areas in Korea.

**Sites**		**Data**	**Formaldehyde**	**Acetaldehyde**	**Acetone+Acrolein**	**Propionaldehyde**	**MEK**	**Ref.**
Road-side	Seoul commercial (2000)	12	8.32	3.38	3.74	0.41	-	[17]
	Gyeongsan commercial (2000)	12	8.43	3.98	4.60	0.42	-	

Urban area	Seoul residential (2000)	12	4.06	2.49	2.14	0.09	-	[17]
	Gyeongsan sub-urban (2000)	12	5.62	3.41	4.34	0.32	-	
	Ulsan sub-urban (2000)	12	5.35	4.35	4.19	0.55	-	
	Seoul residential (2001∼2003)	259	4.61	1.87	4.07	0.17	1.43	[26]
	Seoul residential (2001)	62	4.55	4.01	5.08	0.27	1.54	
	Incheon residential (2002∼2003)	131	4.91	1.89	2.88	0.38	1.18	
	Bucheon suburban (2001)	63	4.59	2.72	3.87	0.23	2.22	
	Gumi commercial (2004)	74	3.36	2.05	2.68	1.03	1.71	This study
	Gumi residential (2004)	75	3.46	2.34	2.74	1.21	1.80	

Industrial area	Siheung industrial (2006)	24	4.13	2.02	1.73	0.47	4.53	[27]
	Ansan industrial (2006)	25	3.92	2.22	2.95	0.41	6.26	
	Yeosu industrial (2008)	80	7.40	2.75	4.05	0.29	2.57	[28]
	Gwangyang industrial (2008)	79	5.99	1.97	2.79	0.14	2.74	
	Gumi the 1st industrial (2003∼2004)	172	3.40	7.35	3.28	0.80	2.40	This study
	Gumi the 2nd industrial (2004)	78	3.87	2.52	4.27	1.01	4.81	
	Gumi the 3rd industrial (2004)	77	3.86	4.22	3.59	1.04	3.32	

**Table 7.** Pearson correlation coefficients between carbonyl compounds and other air quality parameters.

**Sites**	**Compounds**	**SO_2_**	**PM_10_**	**O_3_**	**NO_2_**	**CO**	**Temp. (°C)**	**Wind speed (m/s)**	**R.H. (%)**
1st industrial site (n = 172)	Formaldehyde	−0.05	0.01	−0.12	0.23	−0.01	0.51*	−0.39*	0.28*
	Acetaldehyde	0.24*	0.25	−0.10	0.31*	−0.04	−0.05	−0.17*	0.18*
	Acetone+Acrolein	0.18*	0.17	−0.01	0.40*	0.06	0.12	−0.29*	0.03
	Propionaldehyde	−0.28*	−0.20*	−0.08	0.01	−0.04	0.48*	−0.25*	0.45*
	Methyl ethyl ketone	0.19*	0.11	−0.24*	0.40*	0.21*	−0.32*	0.08	0.12

Commercial site (n = 74)	Formaldehyde	−0.28*	0.10	−0.08	< 0.01	−0.15	0.54*	−0.41*	0.34*
	Acetaldehyde	−0.08	0.10	−0.07	0.05	−0.22	0.53*	−0.47*	0.19
	Acetone+Acrolein	0.32*	0.43*	−0.08	0.31*	0.14	0.01	−0.25*	−0.09
	Propionaldehyde	−0.46*	−0.19	−0.14	−0.11	−0.03	0.47*	−0.26*	0.45*
	Methyl ethyl ketone	0.14	0.49*	−0.01	0.44*	−0.17	0.08	−0.13	−0.21

Residential site (n = 75)	Formaldehyde	0.14	0.01	0.04	0.14	0.02	0.30*	−0.40*	0.06
	Acetaldehyde	0.11	−0.08	0.17	−0.03	0.26*	0.37*	−0.18	−0.03
	Acetone+Acrolein	0.22	0.25*	0.26*	0.29*	−0.03	0.25*	−0.22	−0.35*
	Propionaldehyde	−0.23*	−0.23	−0.15	−0.26*	−0.52*	0.50*	−0.24*	0.41*
	Methyl ethyl ketone	0.24*	0.05	−0.32*	0.12	−0.15	0.48*	−0.15	−0.48*

* Correlation coefficients are significant at a level of 0.05.

**Table 8.** Correlation coefficients between different carbonyl compounds.

**Sampling site**	**Compound.**	**Formaldehyde**	**Acetaldehyde**	**Acetone+Acrolein**	**Propionaldehyde**
1st industrial site (n = 172)	Acetaldehyde	0.21*			
	Acetone+Acrolein	0.41*	0.11		
	Propionaldehyde	0.47*	0.01	0.16*	
	Methyl ethyl ketone	0.03	0.07	0.36*	0.11

2nd industrial site (n = 78)	Acetaldehyde	0.85*			
	Acetone+Acrolein	0.20	0.30*		
	Propionaldehyde	0.53*	0.47*	−0.03	
	Methyl ethyl ketone	0.26	0.33*	0.36*	0.09

3rd industrial site (n = 77)	Acetaldehyde	0.27*			
	Acetone+Acrolein	0.48*	0.63*		
	Propionaldehyde	0.64*	0.03	0.03	
	Methyl ethyl ketone	0.18	−0.05	0.38*	0.03

Commercial site (n = 74)	Acetaldehyde	0.65*			
	Acetone+Acrolein	0.15	0.28*		
	Propionaldehyde	0.60*	0.57*	0.01	
	Methyl ethyl ketone	0.13	0.21	0.45*	0.09

Residential site (n = 75)	Acetaldehyde	0.32*			
	Acetone+Acrolein	0.46*	0.48*		
	Propionaldehyde	0.25*	0.46*	0.06	
	Methyl ethyl ketone	0.10	0.32	0.36*	0.19

* Correlation coefficients are significant at a level of 0.05.
